# Electronic structure and optical properties of doped γ-CuI scintillator: a first-principles study

**DOI:** 10.1039/d2ra07988g

**Published:** 2023-03-24

**Authors:** Meicong Li, Zheng Zhang, Qiang Zhao, Mei Huang, Xiaoping Ouyang

**Affiliations:** a State Key Laboratory of Disaster Prevention & Reduction for Power Grid Transmission and Distribution Equipment, State Grid Hunan Electric Power Company Disaster Prevention and Reduction Center Changsha 410129 China; b Beijing Key Laboratory of Passive Safety Technology for Nuclear Energy, North China Electric Power University Beijing 102206 China qzhao@ncepu.edu.cn +86 10 6177 1665 +86 10 6177 1672; c School of Nuclear Science and Engineering, North China Electric Power University Beijing 102206 China; d Department of Nuclear Physics, China Institute of Atomic Energy Beijing 102413 China; e Northwest Institute of Nuclear Technology Xi'an 710024 China

## Abstract

A cuprous iodide (CuI) crystal is considered to be one of the inorganic scintillator materials with the fastest time response, which is expected to play an important role in the field of γ and X rays detection in the future. To improve the detection performance of the CuI scintillator, the effects of element doping on the electronic structure and optical properties of the γ-CuI were investigated by using the first principles calculation method. It was found that Li and Na doping increases the band gap of the γ-CuI scintillator, while Cs, F, Cl, and Br doping decreases the band gap. The optical absorption coefficient of the γ-CuI scintillator is decreased by the Li and Na doping, and the Cs, F, Cl, and Br doping has little effect on the optical absorption coefficient. The effects of the Tl doping on the electronic structure and optical properties of the γ-CuI scintillator depends on its concentration. Based on the changes in the electronic structure and optical properties, we conclude that the Cs, F, Cl, and Br doping might be a good method that can enhance the detection performance of the γ-CuI scintillator.

## Abstract 2

Element doping can affect the electronic structure and optical properties of γ-CuI. First principles calculations show that Cs, F, Cl, and Br doping may enhance the detection performance of γ-CuI scintillators.

## Graphical abstract

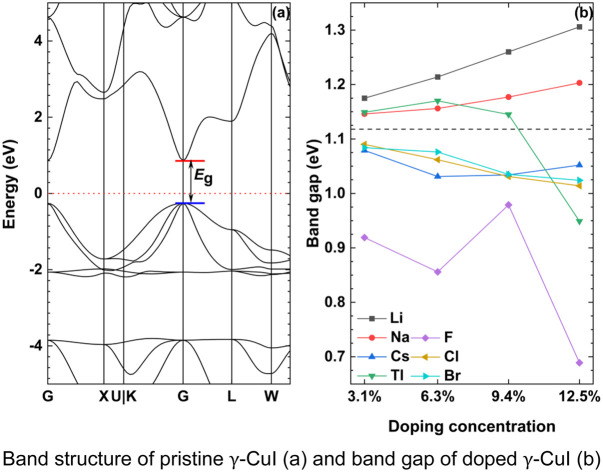

## 1 Introduction

In recent years, nuclear technology has developed rapidly, and ray detection technology is being used more and more widely in daily life and scientific research.^1^ Scintillator materials are a kind of common material for rays detection, which convert the rays (such as X-rays and γ-rays) into ultraviolet or visible light, and then the light is converted into electrical signals through photomultiplier tubes (PMTs), thus the rays are detected. Scintillator materials play an important role in high-energy physics, astrophysics, nuclear physics, space exploration, oil exploration, security inspection, medical imaging, and other related fields.

Since Bädeker introduced the CuI single crystal in 1907,^2^ it has received much attention. CuI has many unique properties, such as wide band gap, negative spin–orbit splitting, large ionicity, anomalous diamagnetic behaviour, and ultrafast scintillation properties with a decay time of about 90 ps. These excellent properties make it a promising new scintillator material for X-ray and γ-ray detection. CuI is now known to be a water-insoluble solid with three crystalline phases:^3^ γ-CuI, β-CuI, and α-CuI. The CuI crystal is in the γ phase when it is in an environment below 350 °C, in the β phase between 350 and 392 °C, and in the α phase above 392 °C. Therefore, the γ-CuI crystal is the most common of the three phases at ambient temperatures among the three phases. Attempts to grow γ-CuI in a conventional water solution or by melting method have been limited. A variety of approaches have been tried to overcome this challenge, including sublimation,^4^ flux,^5^ slow evaporation,^6^ hydrothermal,^7^ oxygen-free cooling,^8^ and sol–gel methods.^3^ However, the production of a large, high quality γ-Cu single crystal remains a challenging task. Further investigation and optimization of crystal growth methods are required. Doped CuI thin films have been synthesised by various techniques various techniques such as pulsed laser deposition (PLD),^9^ spraying method,^10^ electrochemical deposition,^11^ ethanol thermal method,^12^ and laser-assisted molecular-beam deposition.^13^ In general, the γ-CuI crystal growth technology has made great progress in recent years,^14–17^ it is meaningful to develop the cost-effective CuI-based scintillator materials for X-ray and γ-ray detection.

Previous studies have shown that in the scintillation light emitted by the γ-CuI scintillator, the intensity of the ultrafast component is low, while the intensity of the slow component is high.^18^ The characteristics of the γ-CuI scintillator depend on its electronic structure. The optical properties play a crucial role in its detection performance. Therefore, it is important to regulate and control the electronic structure and optical properties of the γ-CuI scintillator to improve its rays detection performance and broaden its application.

The pristine γ-CuI is a p-type semiconductor due to the inherent defect, the Cu vacancy (V_Cu_), in the crystal growth.^19^ To remove this inherent defect, scientists have tried various approaches. Elements doping is widely considered to be the most promising method.^20–22^ New defect states induced by the elements doping ions will affect the band structure, electronic conductivity, and optical properties of the γ-CuI scintillator, so they are very important for the scintillation properties of the γ-CuI scintillator. Several dopants have been considered for doping the γ-CuI scintillator, including metallic and non-metallic elements. The zinc, cadmium, calcium, magnesium, and group-VIA elements doped γ-CuI scintillators were investigated.^20^ It was found that the group-VIA elements doping has little effect on the p-type conductivity of the γ-CuI scintillator.

With the rapid development of computer technology, theoretical simulation is playing an increasingly important role in materials science.^23–27^ Significantly, first principles calculation method is more and more widely used in the research of novel scintillator materials.^28–30^ Several fundamental limits to scintillator performance are being investigated. The prospects for discovering better scintillators are guided by first principles theoretical calculations of the processes active in scintillation.^31^ Using first-principles calculations, Bang *et al.* reported that Tl doping introduces Tl p states inside the band gap to trap the excited electrons in CsI.^32^ McAllister *et al.* studied direct and phonon-assisted Auger recombination in NaI.^33^ Wu *et al.* showed how to search for efficient activators for LaI_3_.^34^ Schleife *et al.* studied the optical properties of four scintillator materials: NaI, LaBr_3_, BaI_2_, and SrI_2_. By solving the Bethe–Salpeter equation for the optical polarization function, they study the influence of excitonic effects on the dielectric and electron-energy loss functions.^35^ Canning *et al.* studied the mechanism of Tl activated halide scintillator materials.^36^ However, few researchers have addressed the effects of elements doping on the electronic structure and optical properties of γ-CuI scintillator through first principles calculation.^37^ It is well known that Tl-doped halide scintillators are among the most widely used γ-ray detector materials for applications in medical imaging, high energy physics and nuclear material detection. In this work, we choose Li, Na, Cs, Tl, and halogens (F, Cl, and Br) as the doping elements. Based on our previous researches,^38–41^ the effects of the doping elements on the electronic structure and optical properties of the γ-CuI scintillator have been investigated by using first principles calculation method.

## 2 Computational method

All the calculations in this paper were carried out with the Vienna *Ab initio* Simulation Package (VASP) code^42,43^ based on the Density Functional Theory (DFT). The Projector-Augmented Wave (PAW) pseudopotentials method was used to describe the interactions between atomic core and valence electrons. The exchange–correlation potential was represented by the Generalized Gradient Approximation (GGA) in the form of the Perdew–Burke–Ernzerhof (PBE) functional.^44^ The electron wave function was expanded in a basis set of plane waves with a kinetic energy cutoff of 400 eV, which was sufficient for the γ-CuI scintillator. The position of all the atoms was fully relaxed in the configuration optimization process, the Hellman–Feynman force was less than 0.02 eV Å^−1^, and electronic iterations convergence was set to 1.0 × 10^−5^ eV. Monkhorst–Pack *k*-point grids of 3 × 3 × 3 and 5 × 5 × 5 were used for the geometry optimization and self-consistent calculations, respectively.

**Fig. 1:**
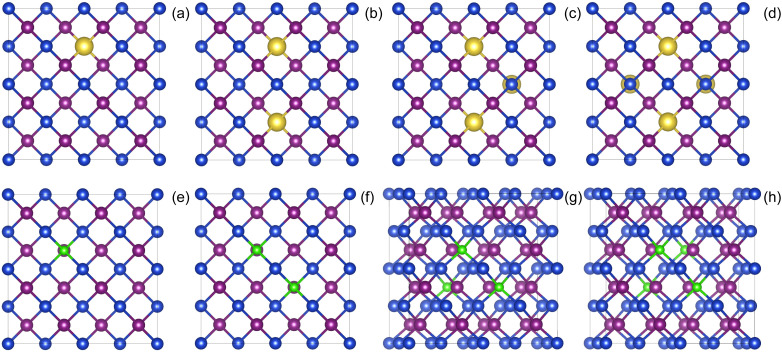
The schematic diagram of the CuI supercell doped with metal (top panels) and nonmetal (bottom panels) elements, and the concentration of doping elements is 3.1% (a and e), 6.3% (b and f), 9.4% (c and g), and 12.5% (d and h). The blue, purple, golden, and green balls are the Cu, I, metal doping elements, and nonmetal doping elements, respectively.

To investigate the effects of elements doping (including Li, Na, Cs, Tl, F, Cl, and Br) on the electronic structure and optical properties of the γ-CuI scintillator, we constructed a 2 × 2 × 2 CuI supercell model containing 32 Cu and 32 I atoms. Based on the results of previous research,^37^ substitutional doping is easily achieved on Cu-site and I-site. In order to eliminate the interaction between the dopant atoms, we have chosen a distance between any two dopant atoms that is much larger than the sum of the radii of the two dopant atoms. In the 2 × 2 × 2 supercell used in the paper, the chosen positions satisfy these requirements and converge in the calculations to obtain a stable structure. We replaced the Cu atoms in the CuI supercell by metal atoms (such as Li, Na, Cs, and Tl), and the I atoms were replaced by non-metal atoms (such as F, Cl, and Br), and at most four atoms in the CuI supercell were replaced by doping elements. The schematic diagram of the doped the γ-CuI scintillator with different doping concentrations is shown in Fig. 1. In this paper, we have used the atomic percentage to represent the doping concentration, and the concentrations of doping elements are 3.1%, 6.3%, 9.4%, and 12.5%, respectively. The electronic structure and density of states were used to investigate the effects of elements doping on the detection performance of the γ-CuI scintillator. To investigate the effects of elements doping on the optical properties of the γ-CuI scintillator, we calculated the optical absorption coefficient and refractive coefficient of the γ-CuI scintillator before and after elements doping.

## 3 Results and discussion

### 3.1 Lattice constant

**Fig. 2:**
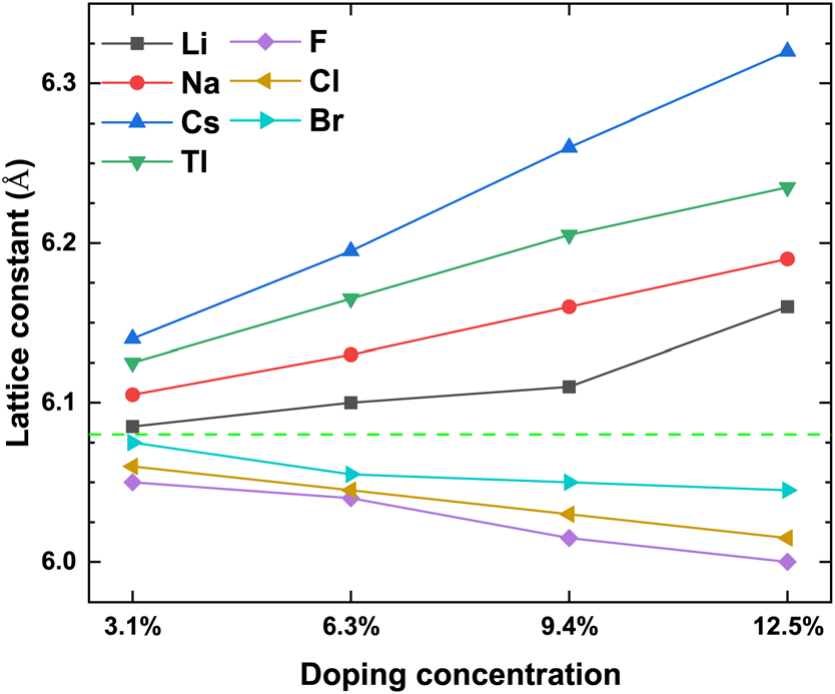
The lattice constant of the CuI unit cell doped with different elements, and the green dash line stands for the lattice constant (6.07 Å) of the pristine CuI unit cell.

**Fig. 3:**
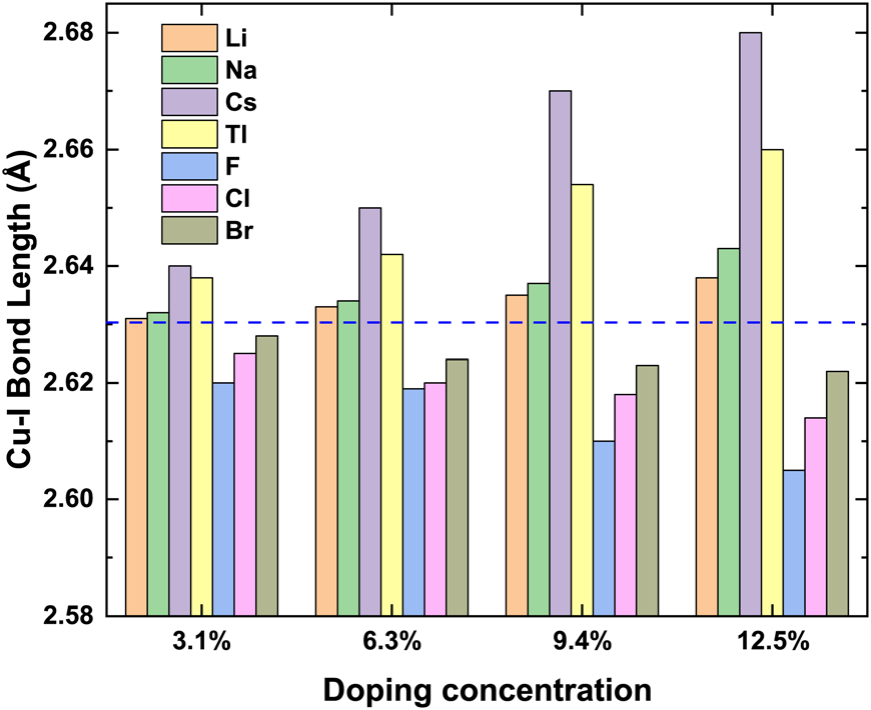
The bond lengths of Cu–I at different concentrations, and the blue dash line stands for average Cu–I bond length (2.63 Å) of the pristine CuI unit cell.

To prove the accuracy of the calculation in this paper, we first calculate the lattice constant of the pristine γ-CuI. The lattice constant value in this work is 6.07 Å as shown in Table 1, which is in good agreement with the previous experimental data^45^ and theoretical results.^46–49^ This shows that our choice of computational details gives good results on structure parameters. The lattice constant of the γ-CuI unit cell doped with different elements is shown in Fig. 2. The lattice constant of the metal elements doped CuI is larger than that of the pristine γ-CuI, and the lattice constant increases with the concentration of metal dopants. The lattice constant of the Cs doped γ-CuI is the largest, followed by Tl and Na doped γ-CuI, and the lattice constant of the Li doped γ-CuI is the smallest. The lattice constant of the non-metal elements doped γ-CuI is smaller than that of the pristine γ-CuI, and the lattice constant decreases with the concentration of the non-metal doping elements. The lattice constant of the F doped γ-CuI is the smallest, followed by Cl and Br doped γ-CuI. The reason for the change in lattice constant is the different atomic radius of the doped atoms, the atomic radius of the metal doping elements is larger than that of the Cu atom, while the atomic radius of the non-metal doping elements is smaller than that of the I atom. The average Cu–I bond lengths at different concentrations are also shown in Fig. 3. The changes in the average Cu–I bond length correspond well with Fig. 2.

**Table 1:** The lattice constant *a* (in Å) of the γ-CuI

Lattice constant	*a*
This work	6.07
Experimental data	6.05 (ref. 45)
Theoretical results	6.09 (ref. 46), 6.097 (ref. 47), 6.073 (ref. 48), 6.05 (ref. 49)

### 3.2 Electronic structure

**Fig. 4:**
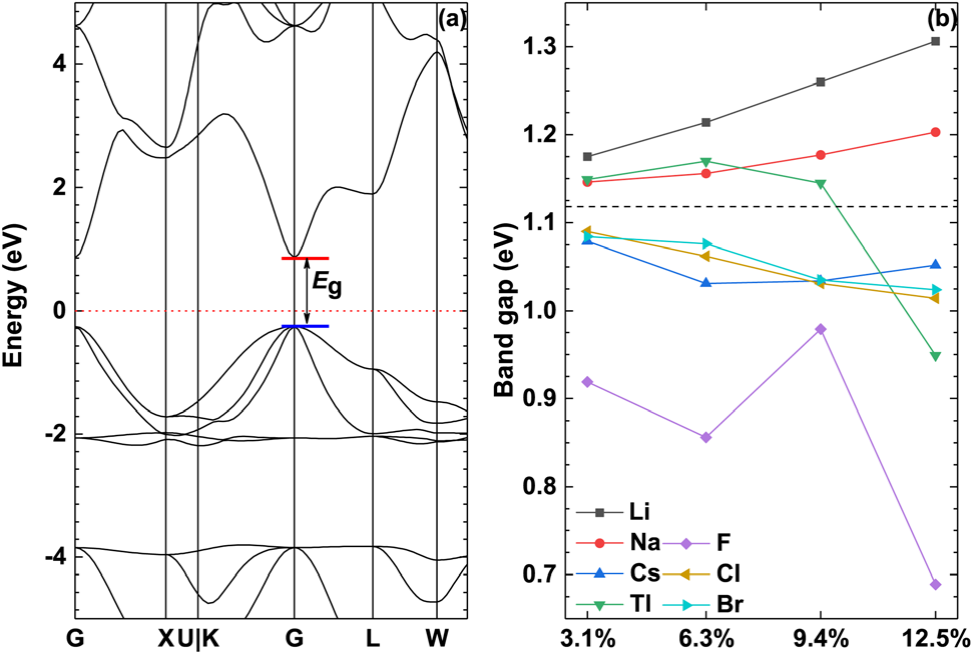
The band structure of the pristine γ-CuI (a) and the band gap of the doped γ-CuI (b), the dashed line represents the band gap of the pristine γ-CuI scintillator, chemical symbol indicates the type of doped element.

Fig. 4 shows the band structure of the pristine γ-CuI scintillator and the band gap of the doped γ-CuI scintillator. It shows that the band gap of the pristine γ-CuI scintillator is 1.118 eV, and this value is in good agreement with previous theoretical results,^46–50^ as shown in Table 2. It should be noted that the band gap of the γ-CuI scintillator calculated by the GGA calculation is lower than the experimental value, because the exchange–correlation interaction between the d and f electrons is not sufficiently described in the GGA calculations. When using the density functional theory calculated by the ordinary plane wave Kohn–Sham equation, the band gap obtained by theoretical calculation is often smaller than the band gap obtained by the actual experiment. In general, DFT + *U* can obtain a band gap consistent with the experiment by adjusting the *U* value, and the hybridization density functional can adjust the hybridization ratio, but these calculation methods require empirical evidence to set the parameters. The GW calculation method consumes a lot of computational resources, and the fully converged GW calculation often obtains a band gap larger than the experimental value. In this paper, we focus on the effect of doping on the band gap of the γ-CuI scintillator, which is the relative energy change rather than the absolute energy.

**Table 2:** The calculated band gap *E*_g_ (in eV) for the pure γ-CuI system compared with experimental data

Band gap	*E*_g_
This work	1.118
Expt. data	3.118 (ref. 51)
GGA	1.05 (ref. 52), 1.12 (ref. 53), 1.165 (ref. 54)
GGA + *U*	1.86 (*U* = 4.8)^55^, 1.89 (*U* = 5.2)^20^, 2.1 (*U* = 6.0)^56^
HSE	2.59 (ref. 48), 2.57 (ref. 37), 3.05 (ref. 55)
GW	1.79 (ref. 57), 2.70 (ref. 58), 3.29 (ref. 53)

To investigate the effect of elements doping on the band gap of γ-CuI scintillator, we calculated the band gap of the doped γ-CuI scintillator as shown in Fig. 4(b). The band gap of the Li and Na doped γ-CuI scintillator is greater than that of the pristine γ-CuI scintillator, the band gap of the Cs, F, Cl, and Br doped γ-CuI scintillator is smaller than that of the pristine γ-CuI scintillator. The band gap of the Tl doped γ-CuI scintillator is greater than that of the pristine γ-CuI scintillator when the doping concentration is less than 9.4%. On the contrary, the band gap of the Tl doped γ-CuI scintillator is smaller than that of the pristine γ-CuI scintillator when the doping concentration is greater than 9.4%. Among the doped γ-CuI scintillators, the Li doped γ-CuI scintillator has the largest band gap, and the F doped γ-CuI scintillator has the smallest band gap. More specifically, the band gap of the Li and Na doped γ-CuI scintillator increases with the concentration, whereas the band gap of the Cl and Br doped γ-CuI scintillator decreases with the concentration. The band gap of the Tl doped γ-CuI scintillator first increases with the concentration and then decreases with the concentration when the concentration is greater than 6.3%. Contrary to the effect of Tl doping, the band gap of the Cs doped γ-CuI scintillator first decreases with the concentration and then increases with the concentration. The band gap of the F doped γ-CuI scintillator fluctuates with the concentration. In short, the Li and Na doping increase the band gap of the γ-CuI scintillator, the Cs, F, Cl, and Br doping decrease the band gap, the effect of Tl doping on the band gap depends on the concentration.

The electronic structure is an important parameter that determines the detection performance of the γ-CuI scintillator. The luminescence spectrum of the γ-CuI scintillator comes from de-excitation after the electrons which are excited by high energy rays, therefore, the luminescence spectrum is closely associated with its band structure. When the γ-CuI scintillator obtains enough energy from the incident rays, electrons can transit from the valence band to the conduction band. At the same time, the transitions leave some holes in the valence band, that is, electron–hole pairs. When an excited electron moves back from the conduction band to the valence band, a photon whose energy is equal to the band gap is produced. The photon yield of the γ-CuI scintillator can be calculated using the following equation:1
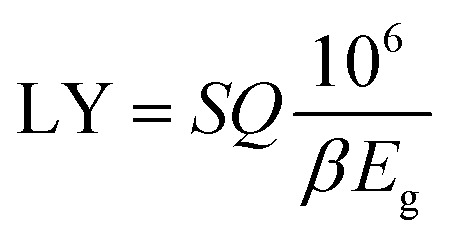
where LY is the photon yield, *E*_g_ is the band gap of the γ-CuI scintillator, *β* is the conversion efficiency (for most semiconductors and insulators, the value is 2–3), *S* is the efficiency of the transfer process, and *Q* is the luminescence quantum efficiency of the center itself, respectively.^59^ For an ultrafast scintillating material with a high count rate such as cuprous iodide, if we consider *S* and *Q* as constants close to 1,^60^ we can draw a conclusion that the smaller the band gap is, the higher the photon yield is. Once the light is produced, there are also some losses during transport to the detector, depending on internal scattering and re-absorption, so the actual light yield of a scintillator can be lower than the theoretically expected value, also depending on the geometry of the scintillator. Theoretically, elemental doping is an effective way to increase photon yields.

According to the eqn (1), the photon yield of the γ-CuI scintillator is decreased by the Li and Na doping, while the Cs, F, Cl, and Br doping increase the photon yield of the γ-CuI scintillator. The Tl doping decreases the photon yield of the γ-CuI scintillator when the concentration is less than 9.4%, and the Tl doping increases the photon yield when the concentration is greater than 9.4%. Some previous theoretical researches^47,50^ have shown that the band gap of the γ-CuCl and γ-CuBr crystals is 0.67 eV and 0.71 eV, respectively. Our result also shows that the band gap of the Cl doped γ-CuI scintillator is smaller than that of the Br doped γ-CuI scintillator. Previous experimental research^61^ has shown that the Cl doping enhances the near-band-edge emission of the γ-CuI scintillator. These previous studies show that the conclusions of this paper are reliable.

**Fig. 5:**
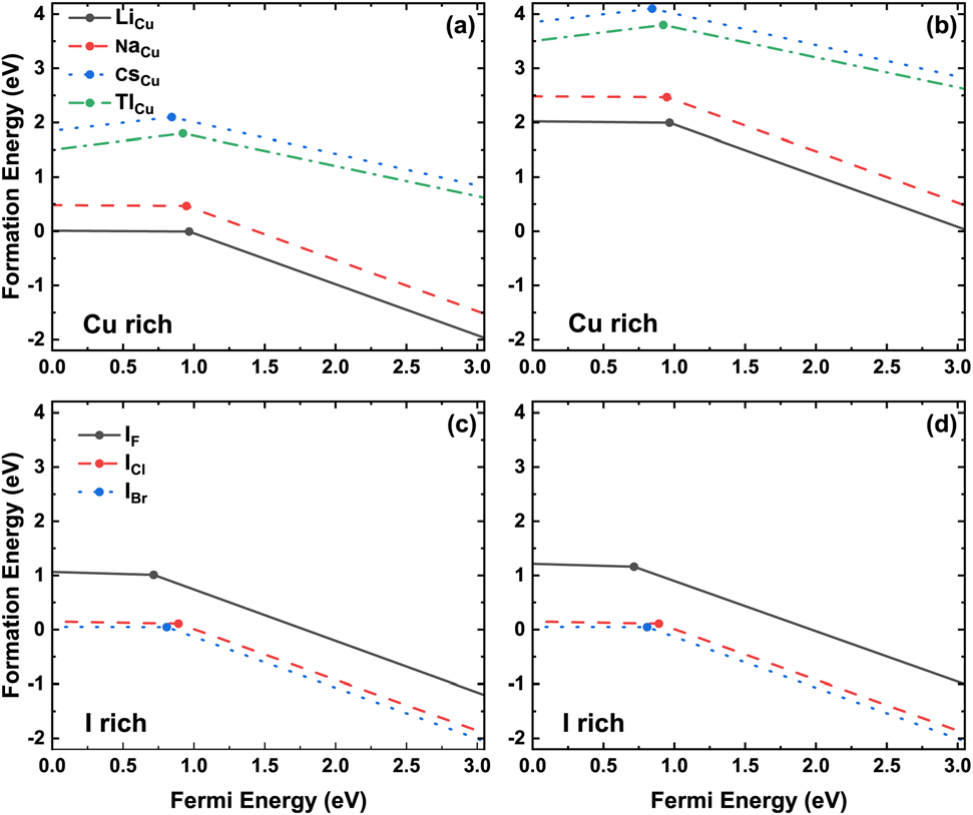
Formation energies of doped defects as function of the Fermi level under the Cu-rich and I-rich limits. The slopes of the line segments represent the defect charge states and the kinks denote the transition energy levels. The concentration of doping elements is 3.1%.

The common method to identify the formation of defects in the crystal structure is to compare the formation energies of various defects, since this parameter is a measure of the defect concentration.^62^ The formation energy of the substitutional defect is calculated as2
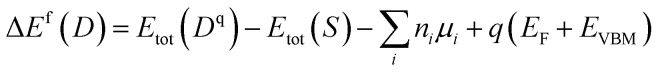
where *E*_tot_(*D*^q^) and *E*_tot_(*S*) refer to the total energy of the system containing the substitutional defects and the defect-free system, respectively. *n*_*i*_ indicates the number of *i*-atoms removed (*n*_*i*_ > 0) or added (*n*_*i*_ < 0), while *μ*_*i*_ is the chemical potential of atom *i*. (*E*_F_ + *E*_VBM_) is the position of the Fermi level relative to the valence band maximum (*E*_VBM_). The Cu-rich and I-rich limits refer to the conditions where the Cu and I chemical potentials reach their maximal values, respectively. We have calculated the formation energies of the subsituational defects in the relevant charge states at a doping element concentration of 3.1%. The results as a function of the Fermi level position for the Cu-rich/I-poor and Cu-poor/I-rich boundaries are shown in Fig. 5. The slopes of the line segments represent the defect charge states and the kinks denote the transition energy levels. The Fermi level at the joint between nearby charge states represents the location of the thermodynamic transition level at the band gap. The formation energies follow Li > Na > Tl > Cs. The transition levels *ε*(0/−) are located at 0.97, 0.95, 0.85, and 0.92 eV above the top of the valence band for Li_Cu_, Na_Cu_, Cs_Cu_, and Tl_Cu_, respectively. The F_I_, Cl_I_, and Br_I_ show the *ε*(−1/−2) at 0.72, 0.89, and 0.81 eV, respectively. The formation energies decrease from F to Br for the substitutional defects due to the ionic radii.

**Fig. 6:**
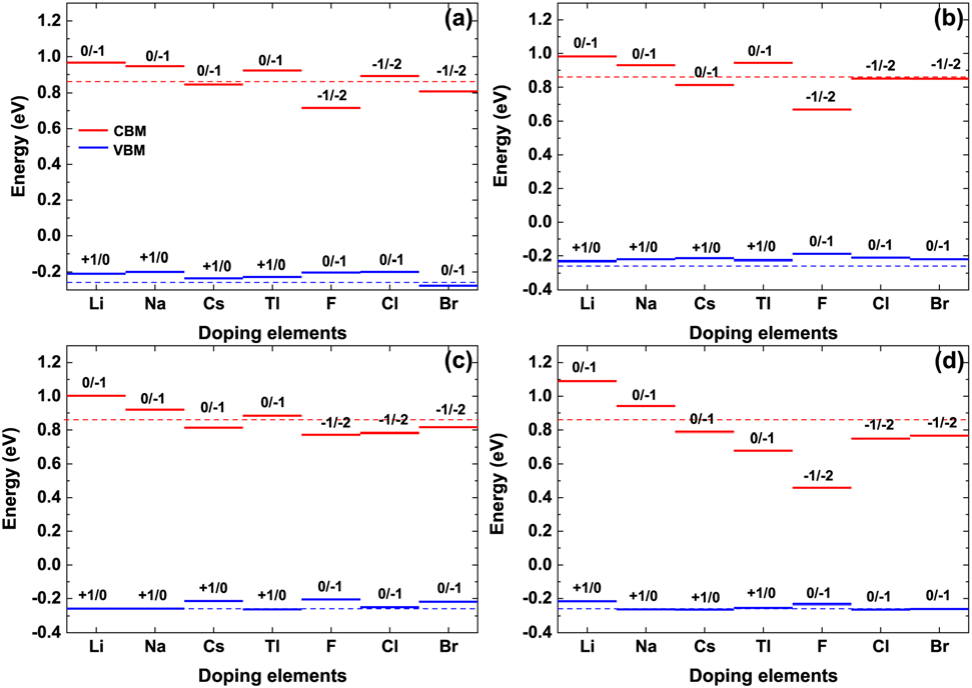
The VBM and CBM of the doped γ-CuI, and the concentration of doping elements is 3.1% (a), 6.3% (b), 9.4% (c), and 12.5% (d). The dashed lines stand for the VBM and CBM of the pristine γ-CuI scintillator.

The valence band maximum (VBM) and conduction band minimum (CBM) of the doped γ-CuI scintillator are shown in Fig. 6. Band gap is the gap between the VBM and CBM, therefore, the band gap change is determined by the position of the VBM and CBM. For the Li, Na, and Tl doped γ-CuI scintillators, both VBM and CBM move toward the direction of energy increase when they are compared with the corresponding value of the pristine γ-CuI scintillator. The band gap of the doped γ-CuI scintillator increases because the CBM moves more than the VBM. The band gap of the Cs, F, Cl, and Br doped γ-CuI scintillator is smaller than that of the pristine γ-CuI scintillator, and this tendency increases with the doping element concentration, the reason for these changes being that the CBM moves in the opposite direction when the VBM moves towards the direction of increasing energy.

**Fig. 7:**
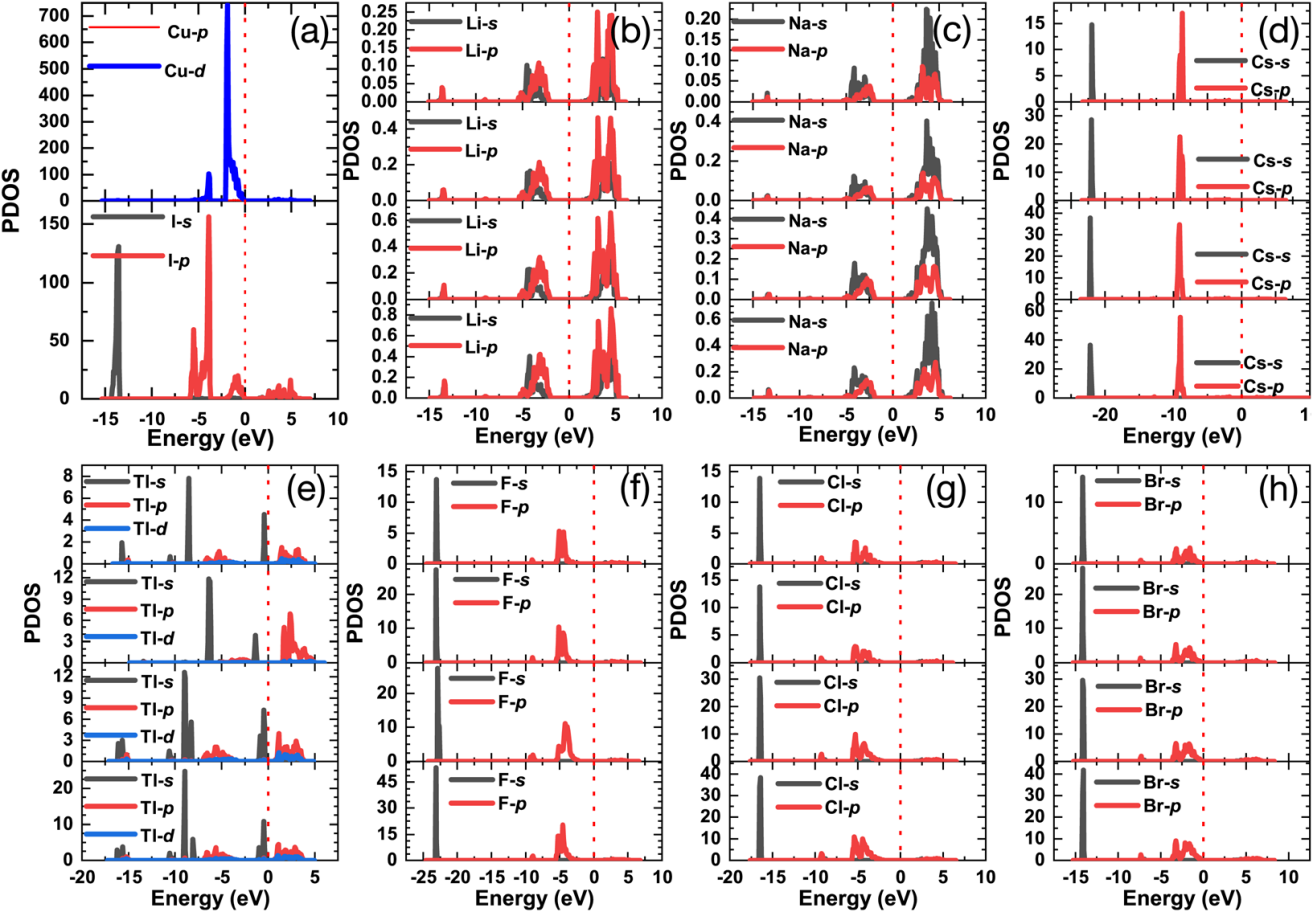
The project density of states of the pristine (a) and Li (b), Na (c), Cs (d), Tl (e), F (f), Cl (g), and Br (h) doped γ-CuI scintillators.

Fig. 7 shows the project density of states (PDOS) of all doped γ-CuI scintillator, which can be used to explore the deep mechanism of the band gap changes of the γ-CuI scintillator. The states near the VBM of the γ-CuI scintillator is composed by the Cu 3d and I 5p orbits, while the states near the CBM are mainly contributed by the I 5p orbit. After the Li, Na, and Tl doping, the band gap of the γ-CuI scintillator increases because there is no impurity level between the VBM and the CBM, and the CBM moves towards the direction of increasing energy. The band gap of the Tl doped γ-CuI scintillator with a concentration of 12.5% is smaller than that of the pristine γ-CuI scintillator because the peak near the CBM moves into the band gap. Some impurity levels appear between the VBM and the CBM after doping, which is caused by the s orbit of Cs, F, Cl, and Br. Therefore, the band gap of the γ-CuI scintillator is decreased by the Cs, F, Cl, and Br doping.

**Fig. 8:**
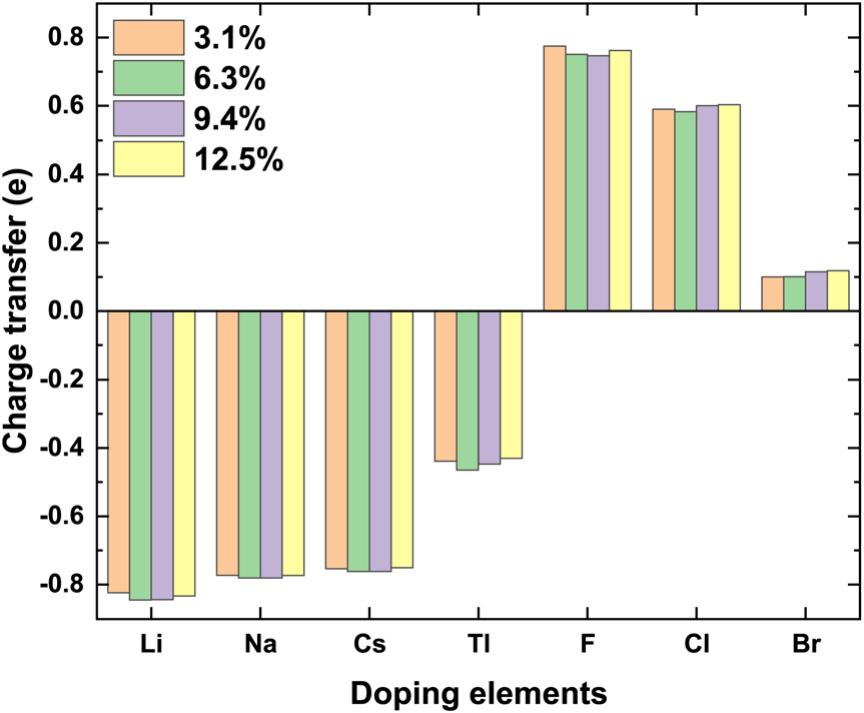
The total charge transfer *Q*_transfer_ of the doping elements in the doped γ-CuI scintillator.

Total charge transfer *Q*_transfer_ represents the charge transfer between the doping elements and their adjacent atoms in the doped γ-CuI scintillator, and the *Q*_transfer_ is calculated by the following equation:

3 *Q*_transfer_ = *Q*_Bader_ − *Q*_ZVAL_

where, *Q*_Bader_ and *Q*_ZVAL_ are the Bader charge and atomic valence of the doping elements, respectively. A negative *Q*_transfer_ means that the charge is transferred from the doping elements to their adjacent atoms, while a positive *Q*_transfer_ means the opposite. The Bader charge of Cu and I atoms in the γ-CuI is 10.69 and 7.31 respectively. There are 0.31*e* transferred from the Cu to the I. As shown in Fig. 8, the *Q*_transfer_ of the Li, Na, Cs, and Tl are about −0.83*e*, −0.78*e*, −0.76*e*, and −0.44*e*, respectively, which means that the charge transferred from the doping elements to their adjacent I atoms is more than that of the Cu. As the electronegativity decreases from Li to Tl, the total charge transferred from the doping atoms to I increases, which is shown as an absolute decrease in the negative values. The *Q*_transfer_ of the F, Cl, and Br are about 0.75*e*, 0.59*e*, and 0.11*e*, respectively. The charge transfer between the doping elements and their adjacent Cu atoms decreases from F to Br because the electronegativity of these doping elements decreases from F to Br. Therefore, the elements Li, Na, Cs, and Tl act as the electron donors in the doped γ-CuI scintillator, while the elements F, Cl, and Br act as the electron acceptors.

### 3.3 Optical properties

As an excellent scintillator, high luminous efficiency and a weakly visible light absorption coefficient are the necessary characteristics.^63^ Overlap between the emission and absorption spectra should be avoided, the greater the distance between them is, the better the detection efficiency of the scintillator is. The optical properties of the γ-CuI scintillator are determined by the frequency-dependent dielectric function:

4 *ε*(*ω*) = *ε*_1_(*ω*) + i*ε*_2_(*ω*)

where, *ε*_2_(*ω*) is the imaginary part which is calculated from the momentum matrix elements between the occupied and unoccupied states with the selection rules, and *ε*_1_(*ω*) is the real part which is derived from the imaginary part *ε*_2_(*ω*) using the Kramers–Kronig dispersion equation.^64^ Based on the frequency-dependent dielectric function, the optical absorption coefficient *α*(*ω*) of the γ-CuI scintillator is calculated by the following equation:5
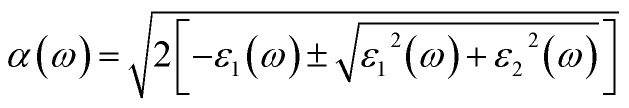
and the optical refractive coefficient *n*(*ω*) is calculated by the equation:6
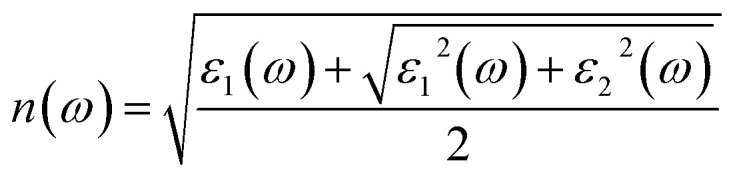


**Fig. 9:**
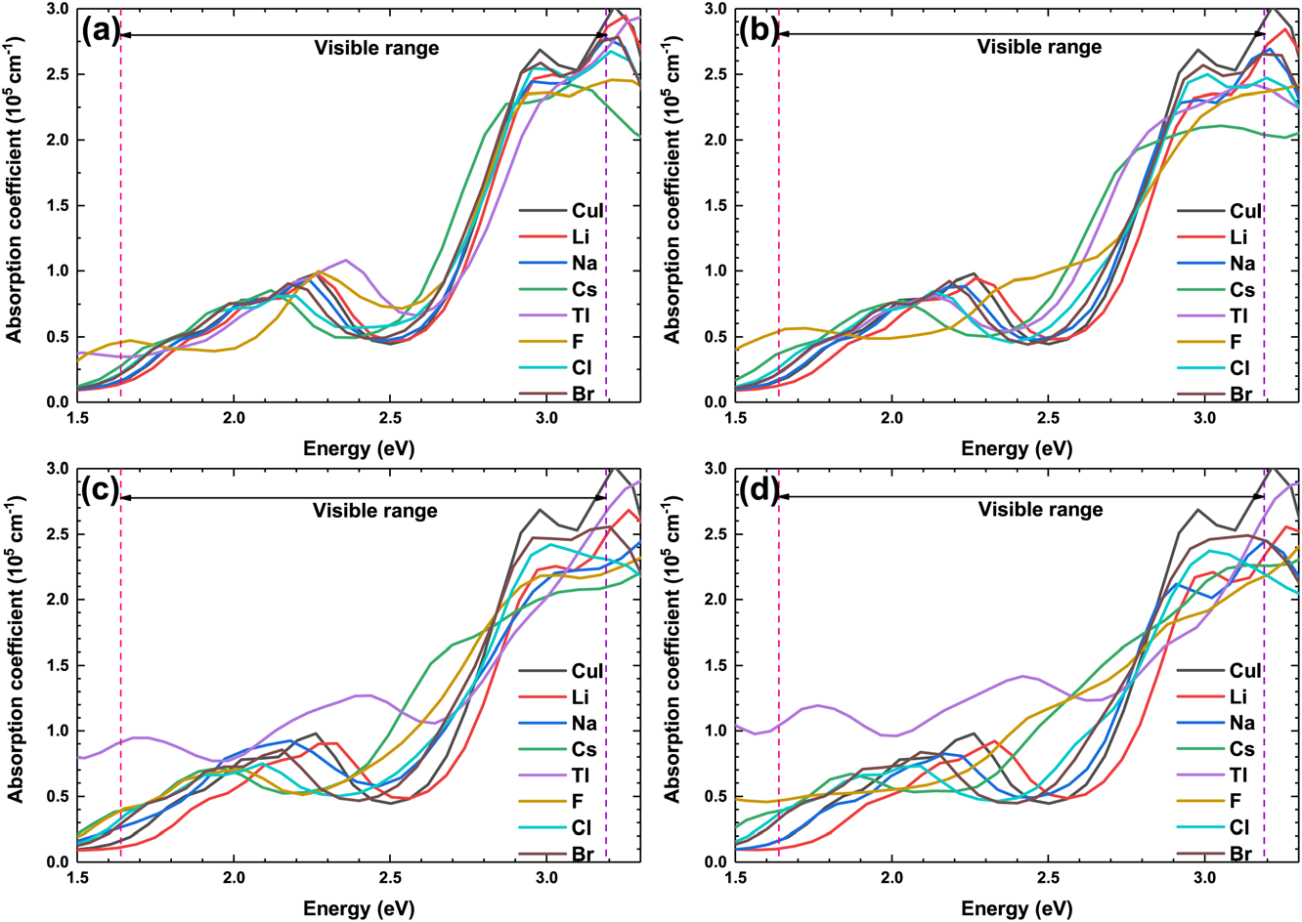
The optical absorption coefficient of the pristine and doped γ-CuI scintillators, and the concentration of doping elements is 3.1% (a), 6.3% (b), 9.4% (c), and 12.5% (d).

Fig. 9 shows the optical absorption coefficient of the pristine and doped γ-CuI scintillator. Due to the best operating range of the photomultiplier is in the visible light range (1.61–3.19 eV), we are more concerned about the effect of elements doping on the optical absorption coefficient of the γ-CuI scintillator in the visible light range. The absorption coefficient of the Li and Na doped γ-CuI scintillator is smaller than that of the pristine γ-CuI scintillator, it means that the absorption of the Li and Na doped γ-CuI scintillator to the visible light is weaker than that of the γ-CuI scintillator. When the concentration of Tl is 3.1% and 6.3%, the absorption of the Tl doped γ-CuI scintillator to the light of 1.61 eV to 2.3 eV is weaker than that of the γ-CuI scintillator, while the absorption of the Tl doped γ-CuI scintillator to the light of 2.3 eV to 2.9 eV is stronger than that of the γ-CuI scintillator. The absorption of the Tl doped γ-CuI scintillator to the visible light is greater than that of the pristine γ-CuI scintillator when the concentration of Tl is 9.4% and 12.5%. In the energy range of 1.61 eV to 2.3 eV, the optical absorption coefficient of Cs, F, Cl, and Br doped γ-CuI scintillator is close to that of the pristine γ-CuI scintillator, which means that elements doping does not significantly change the absorption of visible light in this energy range. The optical absorption coefficient of the Cs, F, Cl, and Br doped γ-CuI scintillator in the energy range from 2.3 eV to 2.9 eV is larger than that of the pristine γ-CuI scintillator, the adsorption of the doped γ-CuI scintillator to the visible light in the energy range from 2.3 eV to 2.9 eV is stronger than that of the pristine γ-CuI scintillator. Except for Br, the band gap of the γ-CuI scintillator decreases with the doping of other elements, the energy of the photons emitted by the doped γ-CuI scintillator decreases, the change in optical absorption coefficient shows that the adsorption of the doped γ-CuI scintillator to low energy visible light decreases. Therefore, the elements doping is a good method for enhancing the detection performance of the γ-CuI scintillator.

**Fig. 10:**
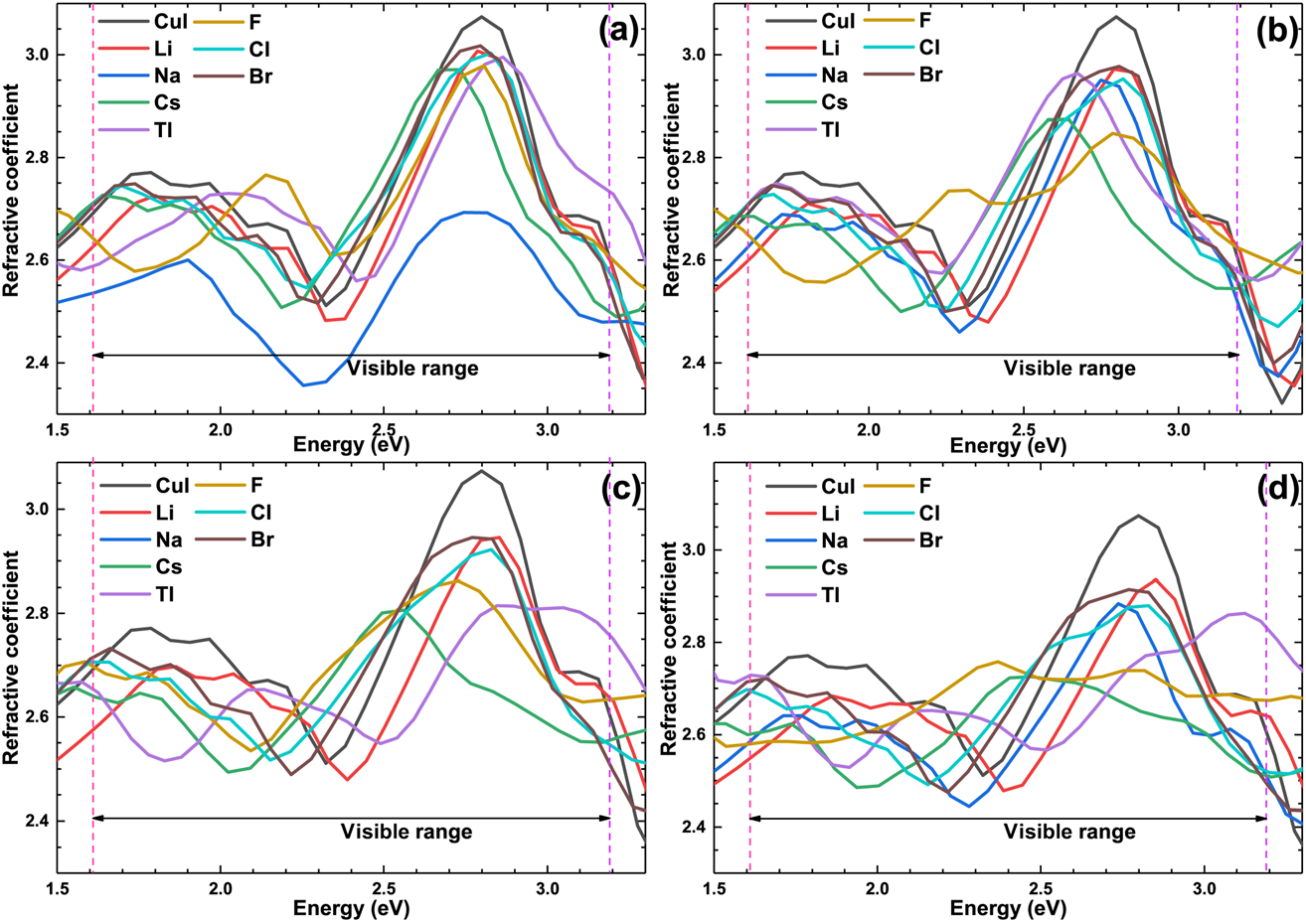
The optical refractive coefficient of the pristine and doped γ-CuI scintillator, and the concentration of doping elements is 3.1% (a), 6.3% (b), 9.4% (c), and 12.5% (d).

Fig. 10 shows the optical refractive coefficient of the pristine and doped γ-CuI scintillator in the visible light energy range. The optical refractive coefficient of the doped γ-CuI scintillator is smaller than that of the pristine γ-CuI scintillator. The binding ability of the anion to the valence electron is enhanced by the elements doping, which makes it more difficult for the outer electrons to be polarized. Therefore, elements doping reduces the optical refractive coefficient of the γ-CuI scintillator.

## 4 Conclusion

To improve the detection performance of the γ-CuI scintillator, the effects of different elements doping on the electronic structure and optical properties of the γ-CuI scintillator were investigated by using first principles calculation method. It found that the Li and Na doping increases the band gap of the γ-CuI scintillator. The band gap of the Tl-doped γ-CuI scintillator is greater than that of the undoped when the concentration is lower than 9.4% and is smaller when the concentration is higher than 9.4%. The Cs, F, Cl, and Br doped γ-CuI scintillator band gap is smaller than that of the γ-CuI scintillator. As a result, the Li and Na doping decrease the photon yield of the γ-CuI scintillator, while the Cs, F, Cl, and Br doping increase the photon yield. Previous experimental research has also shown that the Cl doping can improve the luminescence and scintillating properties of the CuI scintillator. The photon yield of the Tl doped γ-CuI scintillator depends on the Tl concentration, the photon yield is decreased by the Tl doping when the Tl concentration is lower than 9.4%, and the photon yield is increased by the Tl doping when the Tl concentration is higher than 9.4%. The Li and Na doping decrease the optical absorption coefficient of the γ-CuI scintillator in the visible light energy range, while the Cs, F, Cl, and Br doping have little effects on the optical absorption. The optical absorption coefficient of the Tl doped γ-CuI scintillator is close to that of the γ-CuI scintillator when the Tl concentration is 3.1% and 6.3%, and the optical absorption coefficient of the Tl doped γ-CuI scintillator is greater than that of the γ-CuI scintillator when the concentration is higher than 6.3%. Based on the changes in the electronic structure and optical properties, we conclude that the Cs, F, Cl, and Br doping can enhance the detection performance of the γ-CuI scintillator.

## Author contributions

Meicong Li: investigation, methodology, writing – original draft. Zheng Zhang: conceptualization, methodology, writing – original draft. Qiang Zhao: validation, writing – review & editing. Mei Huang: formal analysis, funding acquisition. Xiaoping Ouyang: supervision.

## Conflicts of interest

There are no conflicts to declare.

## Supplementary Material
